# Methylenetetrahydrofolate Reductase (*MTHFR*) C677T Polymorphism and Age at Onset of Schizophrenia: No Consistent Evidence for an Association in the Nordic Population

**DOI:** 10.1002/ajmg.b.32104

**Published:** 2012-10-17

**Authors:** Peter Saetre, Jakob Grove, Anders D Børglum, Ole Mors, Thomas Werge, Ole A Andreassen, Maria Vares, Ingrid Agartz, Lars Terenius, Erik G Jönsson

**Affiliations:** 1 Department of Clinical Neuroscience, Karolinska Institutet and HospitalStockholm, Sweden; 2 Department of Biomedicine, Aarhus UniversityAarhus, Denmark; 3 Bioinformatics Research Centre (BiRC), Aarhus UniversityAarhus, Denmark; 4 deCODE GeneticsReykjavik, Iceland; 5 Center for Psychiatric Research, Aarhus University HospitalAarhus, Denmark; 6 Research Institute of Biological Psychiatry, Mental Health Center Sct. Hans, Copenhagen University HospitalRoskilde, Denmark; 7 Institute of Psychiatry, University of OsloOslo, Norway; 8 Department Psychiatry, Oslo University Hospital—UllevålOslo, Norway; 9 Department of Psychiatry, Section Vinderen, University of OsloOslo, Norway

**Keywords:** methylenetetrahydrofolate reductase gene (*MTHFR*), schizophrenia, age of onset, Scandinavia

## Abstract

Methylenetetrahydrofolate reductase (MTHFR) is an enzyme involved in metabolic pathways of importance for nucleotide synthesis and methylation of DNA, membranes, proteins and lipids. The *MTHFR* gene includes a common polymorphism (rs1801133 or C677T), which is associated with enzyme activity. The T-allele of the C677T polymorphism has been associated with earlier age at onset of schizophrenia in a Scandinavian population, although no association was found in replication attempts in other populations. Extending the study to five Nordic samples consisting of 2,198 patients with schizophrenia, including the original Scandinavian samples, there was no significant association between *MTHFR* C677T polymorphism and age at onset in schizophrenia. The present results do not suggest that the investigated *MTHFR* polymorphism has any significant influence on age at onset of schizophrenia in the Nordic population. © 2012 Wiley Periodicals, Inc.

## INTRODUCTION

Methylenetetrahydrofolate reductase (MTHFR) is an enzyme involved in metabolic pathways of importance for nucleotide synthesis and methylation of DNA, membranes, proteins and lipids [Frankenburg, 2007]. Normal activity of the enzyme regulates folate and methionine pools and reduces plasma levels of homocysteine [Frankenburg, 2007]. Dysfunctional regulation of methionine-homocysteine metabolism has been anticipated to be significant in schizophrenia aetiology [Muntjewerff et al., 2006], and folate deficiency has been linked to disturbed metabolism of serotonin, dopamine and noradrenaline, neurotransmitter abbreviations possibly contributing to schizophrenia development [Bottiglieri et al., 2000].

The *MTHFR* gene includes two common polymorphisms (rs1801133 or C677T; rs1801131 or A1298C) which both alter enzyme activity and homocysteine concentrations [Frosst et al., 1995; van der Put et al., 1998; Lievers et al., 2001; Hazra et al., 2009]. These polymorphisms have been examined for an association with schizophrenia susceptibility, and the C677T polymorphism has been reported to be associated with the disorder in all meta-analyses conducted, including in the SZGene database [Allen et al., 2008; Shi et al., 2008]. Also a recent genome-wide association (GWA) study reported *MTHFR* C677T among 40 polymorphisms associated with schizophrenia susceptibility in the Han Chinese population [Yue et al., 2011]. However, this or linked polymorphisms on chromosome 1p36.3 were not recorded in recent GWA studies conducted in Caucasian populations [Stefansson et al., 2009; Ripke et al., 2011].

The *MTHFR* C677T functional polymorphism, and the metabolism of folate, methionine, and homocysteine have been extensively studied in relation to etiologically complex chronic diseases, and a relationship between *MTHFR* C677T and age at onset have been observed in for example, coronary artery disease, breast cancer, and Parkinson disease [Mager et al., 2005; Lima et al., 2007; Lin et al., 2007]. In an earlier study, we reported that the *MTHFR* 677 T-allele predispose to an earlier age of onset in unrelated patients with schizophrenia of Scandinavian origin and found similar results in a family-based sample from western China [Vares et al., 2010a]. Given these initial results, we invited authors who had analysed either of these *MTHFR* polymorphisms in unrelated patients with schizophrenia to a combined analysis on age at onset. However, we were unable to replicate the findings outside the initial Scandinavian sample [Vares et al., 2010b; Saetre et al., 2011], as was a Dutch research group [Peerbooms et al., 2010].

To determine whether the initial finding in unrelated individuals was restricted to populations of Scandinavian origin, or more likely due to chance alone, we here analyse the relationship between *MTHFR* C677T polymorphism and age at onset in two additional samples of Icelandic and Danish patients with schizophrenia, and combine the results with the previous Scandinavian results in a meta analysis.

## MATERIALS AND METHODS

### Subjects

The Icelandic sample consisted of patients with schizophrenia, who were recruited all over Iceland as previously described [Stefansson et al., 2009]. Briefly, diagnoses were assigned according to Research Diagnostic Criteria [Spitzer et al., 1978] through the use of the Schedule for Affective Disorders and Schizophrenia Lifetime Version [Spitzer and Endicott, 1977]. Age at onset was determined as the first age when psychiatric help was sought or when symptoms started to cause distress or functional impairment, as defined in OPCRIT [Williams et al., 1996].

The sample from Denmark/Aarhus has also been previously described [Demontis et al., 2011]. It is composed of Danish citizens born from May 1981 and onwards who had a schizophrenia diagnosis (F 20) according to the International Classification of Diseases, 10th revision (ICD-10) [World Health Organisation, 1992] in the Danish Psychiatric Central Registry as of May 2007. Age at onset was determined for each patient as the first date of schizophrenia diagnosis in the registry.

The samples from the initial report [Vares et al., 2010a] were collected in Denmark/Copenhagen (DK), Norway (NO), and Sweden (SE) as previously described [Hansen et al., 2007; Jönsson et al., 2008; Kähler et al., 2008]. Affected individuals were diagnosed with schizophrenia (n = 717), schizoaffective disorder (SCA, n = 87), or schizophreniform disorder (SCP, n = 16), according to ICD-10 (DK) [World Health Organisation, 1992] or DSM-III-R/DSM-IV [American Psychiatric Association, 1987; American Psychiatric Association, 1995] (NO and SE). Age at first admission to a psychiatric hospital department served as the measure of disease onset [Vares et al., 2010a]. All individuals in the study were unrelated and of Caucasian origin.

### Genotyping

Genotyping of the Icelandic group using Illumina Human Hap300Beadchip genome-wide arrays was carried out as previously described [Stefansson et al., 2009]. Samples having yield <98%, sex as determined by X chromosome marker heterozygosity different from their reported sex, evidence of non-European ancestry in STRUCTURE runs, or identity with a higher yield sample already included in the study were excluded. Markers with case or control yield <95%, control Hardy–Weinberg (HW) equilibrium *P* < 1 × 10^−5^, or frequency difference between chip types or typing centers with *P* < 1 × 10^−6^ were also excluded. Only samples with yield >90% were included, and the lower yield of each pair of duplicates was removed. Markers had control HW equilibrium *P* values > 0.001, and, in each group, yield in cases and controls was >95%.

In Denmark/Aarhus, DNA was extracted from dried blood spots provided by the Danish Newborn Screening Biobank using the Extract-N-Amp Blood PCR kit (Sigma-Aldrich, St. Louis, MO). DNA was subsequently whole-genome-amplified using the RepliG mini kit (Qiagen Inc., Valencia, CA) and genotyped on the Illumina Infinium HD Human610-Quad BeadChip (Illumina Inc., San Diego, CA, USA) as previously described [Hollegaard et al., 2009]. For each sample, whole-genome-amplification was performed in three separate reactions, which were pooled before genotyping. Samples with call rates <97% were excluded as were markers with call rates <99%.

In the initial Scandinavian sample [Vares et al., 2010a], genomic DNA was extracted from whole blood samples. The *MTHFR* C677T SNP was genotyped at the SNP Technology Platform in Uppsala, Sweden (http://www.genotyping.se), using the Illumina BeadStation 500GX and the 1536-plex Illumina Golden Gate assay (Illumina Inc.) as previously described [Vares et al., 2010a].

### Statistical Analysis

For each study, we estimated the allele association between the C677T polymorphism and age at onset of schizophrenia with a general linear model. In this primary analysis, age at onset was treated as a quantitative trait, and modeled as a function of gender and the number of T alleles (0, 1, 2). The analyses were conducted in parallel, with R for the Icelandic and Danish/Aarhus sample (version 2.9.0 and 2.11.1, respectively) and with SAS/STAT® software (version 9.3) for the previously investigated Scandinavian samples [Vares et al., 2010a].

Meta analysis of the five studies was done with a simple random-effects model with Proc Mixed in the SAS/STAT® software (SAS institute Inc., Cary, NC). In the analysis, the allele association was modeled as a function of the fixed effect of the intercept and the random effect of study. The beta coefficients (regression slopes) for C677T from the primary analyses were used as the observed effect size, and the corresponding squared standard error (SE) was treated as known variance in the diagonal of the variance–covariance matrix R. HW equilibrium was tested using Fisher's exact test as implemented in PEDSTATS [Wigginton and Abecasis, 2005].

## RESULTS

Genotypes from all included studies were in HW equilibrium. The *MTHFR* 677T allele frequency varied from 27% (Sweden) to 36% (Iceland) in the samples (Table 1). The mean age of onset varied between 18.9 years (Denmark/Aarhus) and 27.6 years (Norway) (Table 1).

**TABLE 1:** Association Between the Methylenetetrahydrofolate Reductase (*MTHFR*) C677T (rs1801133) Polymorphism and Age at Onset of Schizophrenia in Five Samples of North European Origin

Country	n	Age at onset		MAF	HW	Allele association			
	
		Mean	SD			Effect	SE	*t*	*P*
Iceland	517	23.6	6.9	0.36	0.64	−0.08	0.45	−0.17	0.86
Denmark/Aarhus	861	18.9	2.8	0.29	0.56	−0.25	0.15	−1.64	0.10
Denmark/Copenhagen^a^	406	27.2	8.9	0.31	0.73	−1.80	0.66	−2.72	0.007
Norway^a^	159	27.6	8.7	0.32	0.72	−0.94	1.39	−0.91	0.36
Sweden^a^	255	*26.1*	*7.2*	0.27	0.75	*−0.26*	*0.73*	*−0.36*	*0.72*
All studies	2,198					*−0.42*	*0.26*	*−1.65*	*0.17*

Age at onset, minor allele frequencies (MAF), test of Hardy–Weinberg equilibrium (HW; *P*-values) and association statistics are listed for all five studies in a meta-analysis (see text for details)

a Previously included in Vares et al. [2010a].

We have previously reported an association between *MTHFR* C677T polymorphism and age at onset of schizophrenia in a combined Scandinavian sample [Vares et al., 2010a]. When the Danish, Norwegian, and Swedish samples from our previous report were analysed separately we noted that the association signal primarily originated from the Danish (Copenhagen) sample, where the age at onset decreased with on average 1.8 years per T-allele (*P* = 0.007). A negative association between 677T and earlier age at onset was also apparent in the Norwegian sample, although in this sample the association strength was weaker (0.9 years per T-allele, Table 1).

**FIG. 1:**
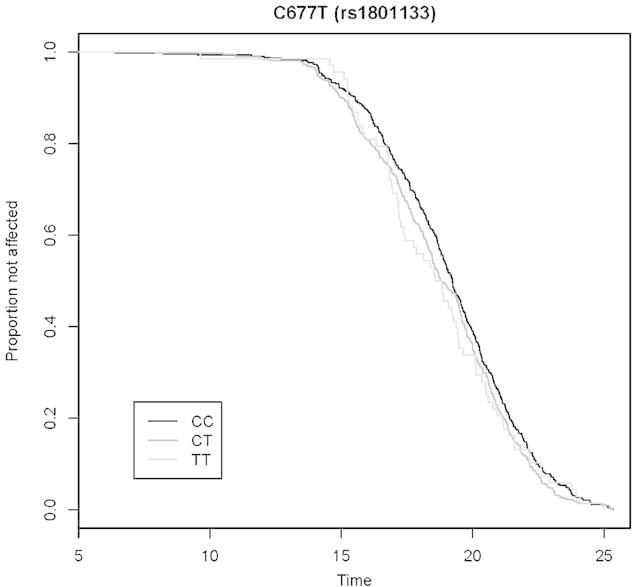
Kaplan-Meier survival curves, describing the proportion of patients who have not yet been affected by schizophrenia, as a function of methylenetetrahydrofolate reductase (*MTHFR)* C677T (rs1801133) genotype (*P* = 0.09) in the Danish (Aarhus) replication sample. The black line represents the CC genotype, dark gray the CT genotype, and light gray the TT genotype, respectively.

In the independent Danish sample (Aarhus), the age at onset decreased with on average 0.25 years per *MTHFR* 677T-allele, but this tendency did not reach statistically significance (*P* = 0.097). We noted that this sample consisted of patients with an earlier age of onset than the other Nordic samples, and most patients became ill before the effect of the *MTHFR* C677T polymorphism was apparent in the original Scandinavian samples [Vares et al., 2010a]. Nevertheless, an inspection of the corresponding Kaplan–Meyer survival plot did not reveal any tendency towards an increased effect of the T-allele with age, as noted in the original study (Fig. 1). We found no association between the *MTHFR* C677T polymorphism and age at onset of schizophrenia in the Icelandic sample (*P* = 0.86). Thus the initial finding of an age dependent association between the *MTHFR* C677T polymorphism and age at onset of schizophrenia in the Scandinavian population could not be replicated in two large and independent samples of similar origin. A meta-analysis of all five samples resulted in a pooled association estimate of 0.42 ± 0.26 years earlier age at onset of schizophrenia per T-allele (mean ± SE), which did not reach statistical significance (*P* = 0.17).

## DISCUSSION

In the present study, we were unable to replicate our original finding of a negative association between the *MTHFR* 677T-allele and age at onset of schizophrenia in two independent samples of northern European heritage (Denmark and Iceland), or in meta-analysis including the initial Scandinavian samples. These results are in line with earlier analyses outside Scandinavia [Peerbooms et al., 2010; Saetre et al., 2011], and recent GWA studies analysing age at onset of schizophrenia [Wang et al., 2011] and bipolar disorder [Belmonte Mahon et al., 2011], which shares genetic susceptibility with schizophrenia.

The association between *MTHFR* 677T-allele and earlier age at onset in our original Scandinavian sample was primarily due to the Danish (Copenhagen) sample, and a similar tendency was present in the Danish (Aarhus) replication sample. Age at onset varied among the cohorts included in the study, and part of this variation may have been related to the different definitions of age at onset used (e.g. start of symptoms, time for first diagnosis, or first admission to a psychiatric clinic). The heterogeneity in the definitions of age at onset is likely to have increased phenotype variation (and thus decreased the power to detect a true but weak association), but we observed no relationship between the definition of age at onset and association strength, neither in this nor in our previous analysis of multiple samples [Saetre et al., 2011]. However, there was a systematic difference in the date of birth of patients included in the studied samples. For example, the majority of patients included in the Danish Copenhagen population were born in the 1960s or earlier, whereas the patients in the Danish (Aarhus) sample were born in the 1980s or later. Thus it is possible that heterogeneity in association strength between the Danish samples were due to a differential effect of the *MTHFR* polymorphism with respect to varying environmental conditions, such as availability of folic acid in food and/or practices of folic acid supplementation during pregnancy. Moreover, the power to detect an effect of the *MTHFR* 677T allele on late onset schizophrenia would be limited in the Danish (Aarhus) sample simply because of its younger age structure. Thus we cannot exclude the possibility of a weak negative association in populations of North European origin.

However, given the data at hand, and previous analysis of samples of different geographical and ethnical origins [Peerbooms et al., 2010; Saetre et al., 2011; Wang et al., 2011], a parsimonious interpretation is that our initial findings [Vares et al., 2010a] were due to chance alone, and that the *MTHFR* 677T-allele has no significant effect on the age at onset of schizophrenia in the general population, irrespective of the geographical location, ethnicity or genetic background. Nevertheless, given the important role of MTHFR in the metabolism on folic acid, methionine, and homocysteine, it is possible that *MTHFR* gene variation not only affects schizophrenia susceptibility but may also modify symptom severity [Herran et al., 1999; Goff et al., 2004] and the efficacy of folic acid supplementation and antipsychotic treatments [Joober et al., 2000; Hill et al., 2011; Vehof et al., 2012].
